# Supplementary material for: Calcium Induces the Cleavage of NopA and Regulates the Expression of Nodulation Genes and Secretion of T3SS Effectors in Sinorhizobium fredii NGR234
Source: Int J Mol Sci. 2024 Mar 19;25(6):3443. doi: 10.3390/ijms25063443 (PMC10970691; doi:10.3390/ijms25063443)
Supplement: Supplementary file 1 [file ijms-25-03443-s001.zip › Supplementary Materials.pdf]

Supplementary Materials

Supplementary Figure 1. Histograms showing band intensity of Nops. Band volume of Nops were measured by densitometer scan of stained SDS-PAGE gel. Band raw volume was obtained using GelAnalyzer software v. 19.1. For comparison the band volume of molecular weight markers are also shown.

Histogram of Lane 1 from Figure 1, showing band intensity.

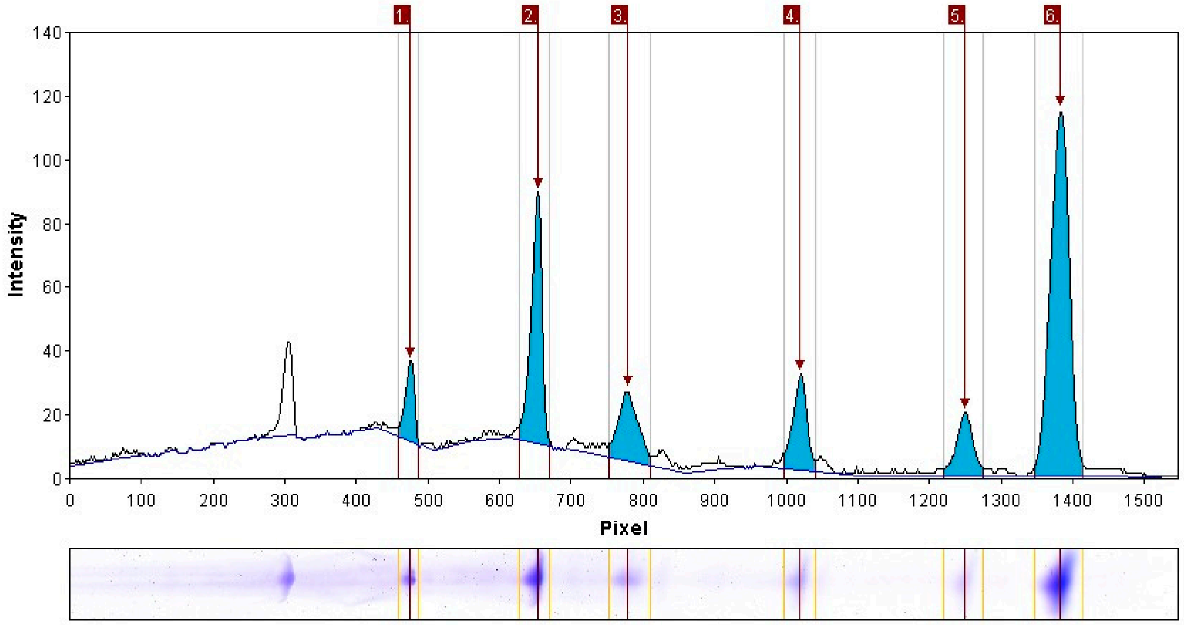

Histogram of Lane 6 from Figure 1, showing band intensity.

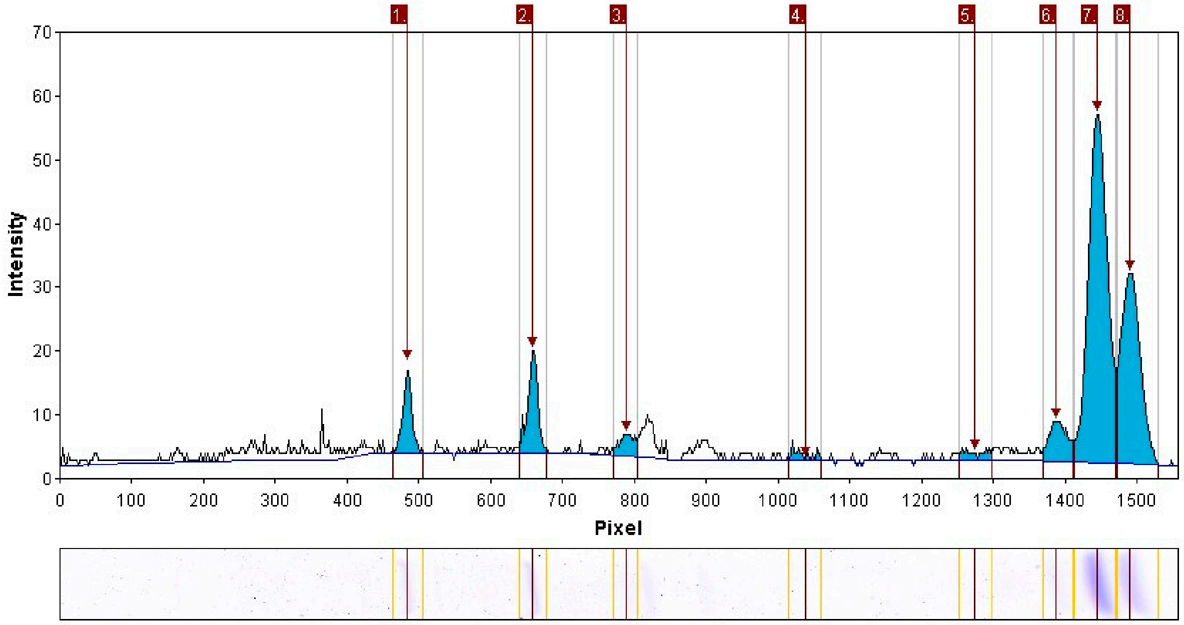

Histogram of Lane M from Figure 1 (molecular weight marker), showing band intensity.

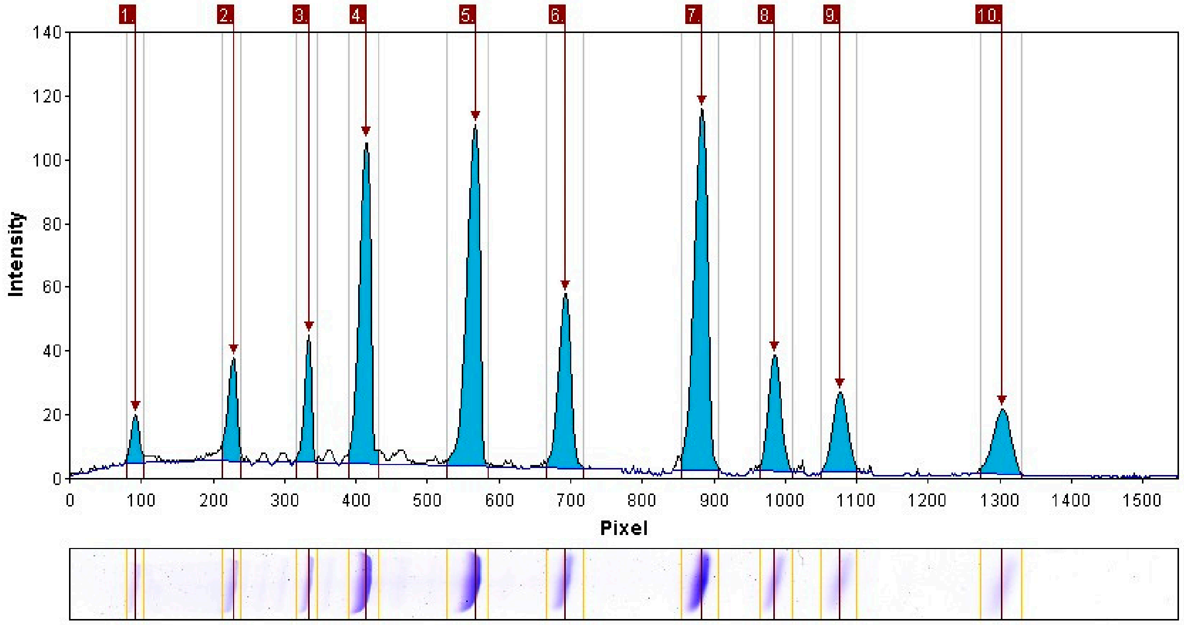

Band volume of Nops measured by densitometer scan of stained SDS-PAGE gel. Band raw volume was obtained using GelAnalyzer software v. 19.1. For comparison the band volume of molecular weight markers are also shown.

**Band #**	**Nop Protein**	**Lane 1 Volume**	**Lane 6 Volume**		*MW*	*Marker Volume*
**1**	**NopX**	**381**	**193**		*250*	*202*
**2**	**NopL**	**1425**	**265**		*150*	*474*
**3**	**NopP**	**704**	**88**		*100*	*476*
**4**	**NopB**	**691**	**39**		*75*	*1893*
**5**	**NopC**	**538**	**53**		*50*	*2316*
**6**	**Nop A**	**3572**	**183**		*37*	*1087*
**7**	**Nop A product**	**---**	**1687**		*25*	*2468*
**8**	**Nop A product**	**---**	**959**		*20*	*777*
					15	*625*
					10	*570*

Supplementary Figure 2. Venn diagram of the up-regulated sets of the DEGs found in the *S. fredii* NGR234 upon treatments with apigenin, calcium, and calcium+apigenin. Apigenin, calcium, and apigenin+calcium treatment showed 31, 11, and 38 exclusive DEGs, respectively. The shared DEGs were 95 to apigenin and apigenin+calcium treatments, 23 to calcium and apigenin+calcium, and only 5 to the three conditions.

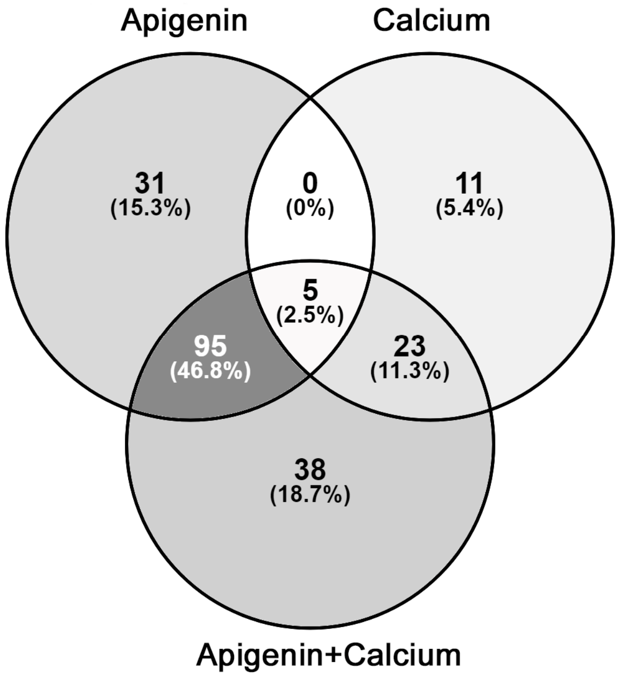

Supplementary Figure 3. Venn diagram of the down-regulated sets of the DEGs found in the *S. fredii* NGR234 upon treatments with apigenin, calcium, and calcium+apigenin. Apigenin, calcium, and apigenin+calcium treatment showed 11, 8, and 7 exclusive DEGs, respectively. The shared DEGs were 10 to calcium and apigenin+calcium treatments, and 5 to the three conditions. The apigenin and apigenin+calcium treatments did not share any DEG.

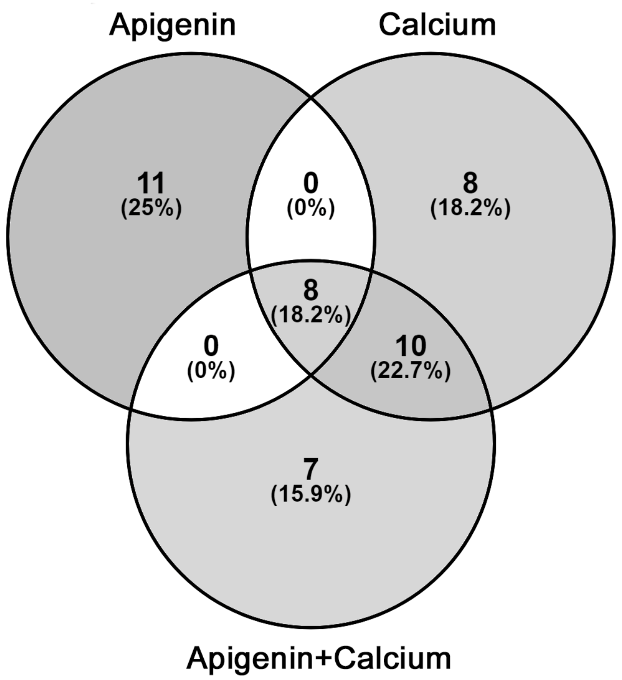

Supplementary Figure 4. Venn diagram of the up-regulated sets of the DEGs found in the *S. fredii* NGR234 upon treatments with apigenin and calcium+apigenin. Apigenin+calcium treatment showed 61 exclusive DEGs, 31 to apigenin treatment, and the two conditions share 100 DEGs.

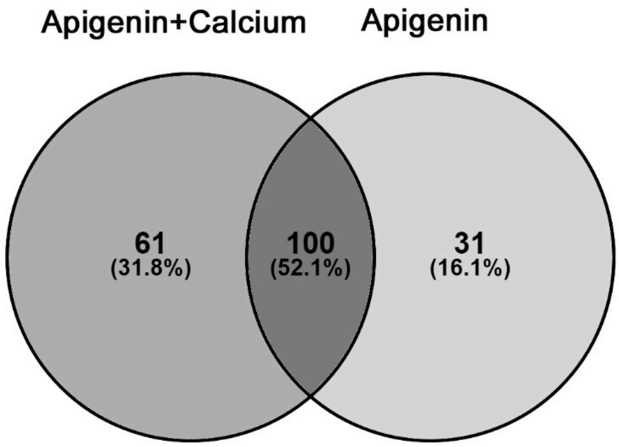

Supplementary Table 1. Primers used in this work.

Oligonucleotide	Sequence 5'-3'	Target
NGR_a03410_fw<br>NGR_a03410_rev	GTTCAACGCAAGACCATTTGA<br>CATCGCCCACCCTGATAAA	Amplification of part of *nodA*
NGR_a00550_fw<br>NGR_a00550_rev	AGAACGTAGCGGCCAAAG<br>GTAACAGTGCGCAGTAGCATA	Amplification of part of *nopA*
NGR_a00670_fw<br>NGR_a00670_rev	AGCTCTGCTGCCTAAGATAAAG<br>CCAAGTCAGTCGCCAGTAAA	Amplification of part of *rhcJ*
NGR_a00470_fw<br>NGR_a00470_rev	CTCGAGGCCTTATTGCAGTATC<br>ATGTCACGCAGCCTTGATAG	Amplification of part of *syrM2*
NGR_a00800_fw<br>NGR_a00800_rev	TAAGGAGCGACGGGTATTCA<br>TAGAGACGGGTTTACGGAGAAA	Amplification of part of *ttsI*
NGR_b17530_fw<br>NGR_b17530_rev	ATGAAGGCCAACCAGGAAG<br>GGTCGTCGCTGTCGAAAT	Amplification of part of transcriptional regulator
NGR_b20640_fw<br>NGR_b20640_rev	TTTGACTGTGGCGCTTCT<br>TGTTGAGCAGGATCGATGTC	Amplification of part of *TRAP-T* family transporter
NGR_c19850_fw<br>NGR_c19850_rev	CTGATCTGCACCAACACCAT<br>CCTCTTTCCTTGAGACGAAGTG	Amplification of part of *asr*
NGR_b03240_fw<br>NGR_b03240_rev	CGTGCTGGACCTCAATCTG<br>Reverse<br>GACGACGACGATCGGAATTT	Amplification of part of *LuxR* family transcriptional regulator
NGR_c31220_fw<br>NGR_c31220_rev	CATGCTTCCGAGATGTTCCA<br>CTTGTGGGCGAGCATCTT	Amplification of part of *lpdA1*
NGR_c17830_fw<br>NGR_c17830_rev	GGATGTATTCGAGAGCGTCTTT<br>CTTGAACATTGCTGCCCTTG	Amplification of part of *sugar ABC* transporter substrate-binding protein
NGR_c30940_fw<br>NGR_c30940_rev	TCATGTTCGCCATCCCTTAC<br>CTCGCCGATTTGCAGAGATA	Amplification of part of *glpD2*
NGR_c07820_fw<br>NGR_c07820_rev	CCAAGGATCTCCGTTTGCT<br>GCAGCACGAACTCACCATA	Amplification of part of *cycL*
NGR_a00400_fw<br>NGR_a00400_rev	CCTGCGATTGAATGTGTTTACC<br>GTTGGCCTCGACATCATCTT	Amplification of part of *nodZ*
NGR_b18360_fw<br>NGR_b18360_rev	AAGCTACCCGGACGACTATT<br>TGCTGAACCCTTCGGTTTG	Amplification of part of *exoA*
NGR_c09220_fw<br>NGR_c09220_rev	AGATGGTTTACGGTGGTGAAG<br>TATACGAGGACCGGGTTCAT	Amplification of part of outer membrane protein
NGR234_16Sr_fw<br>NGR234_16Sr_rev	GCACGTAGGCGGACATTTAAGTCA<br>G<br>GCCACTGGTGTTCCTCCGAATATC	Amplification of part of 16S ribosomal RNA

**Supplementary Table 2**. Number of total and mapped reads for replicates in each condition.							
	**replicon**	**Seq length**	**Total Reads**	**Mapped Reads**	**Unmapped Reads**	**%mapped Reads**	**%unmapped Reads**
Wild-type_1	pNGR234a (U00090.2)	536165	804828	801701	3127	99.61	0.39
	pNGR234b (CP000874.1)	2430033	18596888	18523899	72989	99.61	0.39
	Chromosome (CP001389.1)	3925702	30234000	30112211	121789	99.60	0.40
	**Total/Mean**		**49635716**	**49437811**	**197905**	**99.61**	**0.39**
Wild-type_2	pNGR234a (U00090.2)	536165	855624	852407	3217	99.62	0.38
	pNGR234b (CP000874.1)	2430033	20411653	20348312	63341	99.69	0.31
	Chromosome (CP001389.1)	3925702	32784939	32674113	110826	99.66	0.34
	**Total**		**54052216**	**53874832**	**177384**	**99.66**	**0.34**
Wild-type_3	pNGR234a (U00090.2)	536165	907197	903790	3407	99.62	0.38
	pNGR234b (CP000874.1)	2430033	20940958	20874973	65985	99.68	0.32
	Chromosome (CP001389.1)	3925702	35870197	35746948	123249	99.66	0.34
	**Total**		**57718352**	**57525711**	**192641**	**99.66**	**0.34**
Wild-type_Ap_1	pNGR234a (U00090.2)	536165	2209875	2200835	9040	99.59	0.41
	pNGR234b (CP000874.1)	2430033	14178113	14100560	77553	99.45	0.55
	Chromosome (CP001389.1)	3925702	23898500	23760701	137799	99.42	0.58
	**Total**		**40286488**	**40062096**	**224392**	**99.49**	**0.51**
Wild-type_Ap_2	pNGR234a (U00090.2)	536165	2140510	2132140	8370	99.61	0.39
	pNGR234b (CP000874.1)	2430033	13991992	13918012	73980	99.47	0.53
	Chromosome (CP001389.1)	3925702	22984762	22860546	124216	99.46	0.54
	**Total**		**39117264**	**38910698**	**206566**	**99.51**	**0.49**
Wild-type_Ap_3	pNGR234a (U00090.2)	536165	4725217	4710307	14910	99.68	0.32
	pNGR234b (CP000874.1)	2430033	30538295	30414924	123371	99.60	0.40
	Chromosome (CP001389.1)	3925702	50863134	50648487	214647	99.58	0.42
	**Total**		**86126646**	**85773718**	**352928**	**99.62**	**0.38**
Wild-type_Ca_1	pNGR234a (U00090.2)	536165	634856	631940	2916	99.54	0.46
	pNGR234b (CP000874.1)	2430033	15821383	15740104	81279	99.49	0.51
	Chromosome (CP001389.1)	3925702	26281153	26135572	145581	99.45	0.55

	**Total**		**42737392**	**42507616**	**229776**	**99.49**	**0.51**
Wild-type_Ca_2	pNGR234a (U00090.2)	536165	619699	616867	2832	99.54	0.46
	pNGR234b (CP000874.1)	2430033	15490505	15408323	82182	99.47	0.53
	Chromosome (CP001389.1)	3925702	25539792	25393213	146579	99.43	0.57
	**Total**		**41649996**	**41418403**	**231593**	**99.48**	**0.52**
Wild-type_Ca_3	pNGR234a (U00090.2)	536165	650355	647457	2898	99.55	0.45
	pNGR234b (CP000874.1)	2430033	16553146	16469024	84122	99.49	0.51
	Chromosome (CP001389.1)	3925702	27142679	26994394	148285	99.45	0.55
	**Total**		**44346180**	**44110875**	**235305**	**99.50**	**0.50**
Wild-type_ApCa_1	pNGR234a (U00090.2)	536165	3535006	3524321	10685	99.70	0.30
	pNGR234b (CP000874.1)	2430033	16238057	16186563	51494	99.68	0.32
	Chromosome (CP001389.1)	3925702	32575707	32461574	114133	99.65	0.35
	**Total**		**52348770**	**52172458**	**176312**	**99.68**	**0.32**
Wild-type_ApCa_2	pNGR234a (U00090.2)	536165	3399174	3389120	10054	99.70	0.30
	pNGR234b (CP000874.1)	2430033	16152618	16103556	49062	99.70	0.30
	Chromosome (CP001389.1)	3925702	28245060	28150024	95036	99.66	0.34
	**Total**		**47796852**	**47642700**	**154152**	**99.69**	**0.31**
Wild-type_ApCa_3	pNGR234a (U00090.2)	536165	3583779	3572001	11778	99.67	0.33
	pNGR234b (CP000874.1)	2430033	17304645	17246990	57655	99.67	0.33
	Chromosome (CP001389.1)	3925702	30264642	30153312	111330	99.63	0.37
	**Total**		**51153066**	**50972303**	**180763**	**99.66**	**0.34**

Supplementary Table 3. Mass spectrometric identification of amino acid sequences of NGR234 low molecular weight proteins isolated from acrylamide gels.

Protein ID<br>----<br>Accession	Mascot Score	Peptides (Unique Spectra)<br>----<br>% Coverage	MW Thr. / Exp.	(Position)	Peptide Matched
Band A<br>Nodulation protein NopA<br>(*Sinorhizobium fredii*)<br>----<br>AY775562.2<br>(gi|499177556)	1536	8 (21)<br>----<br>100%	7,004 / 7,000	(1-25)<br>(3-25)<br>(5-25)<br>(24-49)<br>(31-49)<br>(48-64)<br>(53-64)<br>(64-71)	(-)MSKIGTLTSAVGAGAAAGQNVAAK(G)<br>(K)IGTLTSAVGAGAAAGQNVAAK(G)<br>(G)TLTSAVGAGAAAGQNVAAK(G)<br>(K)GAGAAAFQAQIAELAAVSAEATAR(S)<br>(F)QAQIAELAAVSAEATAR(S)<br>(R)SMLLRTVTTELQTTK(K)<br>(R)TVTTELQTTK(K)<br>(K)AADERVQ(-)
Band B<br>Nodulation protein NopA<br>(*Sinorhizobium fredii*)<br>----<br>AY775562.2<br>(gi|499177556)	1061	4 (12)<br>----<br>85%	7,004 / 4,000	(3-25)<br>(29-49)<br>(53-64)<br>(64-71)	(K)IGTLTSAVGAGAAAGQNVAAK(G)<br>(A)AFQAQIAELAAVSAEATAR(S)<br>(R)TVTTELQTTK(K)<br>(K)AADERVQ(-)
Band C<br>Nodulation protein NopA<br>(*Sinorhizobium fredii*)<br>----<br>AY775562.2<br>(gi|499177556)	465	4 (7)<br>----<br>87%	7,004 / 3,000	(3-25)<br>(29-49)<br>(48-64)<br>(64-71)	(K)IGTLTSAVGAGAAAGQNVAAK(G)<br>(A)AFQAQIAELAAVSAEATAR(S)<br>(S)MLLRTVTTELQTTK(K)<br>(K)AADERVQ(-)
